# 

**Published:** 1836-01-01

**Authors:** 


### CONTENTS

OF THE

### MEDICO-CHIRURGICAL REVIEW,

### No. XLVII. JANUARY 1, 1836.

[BEING No. VII. OF A DECENNIAL SERIES.]

### REVIEWS.

I.

PATHOLOGICAL ANATOMY.

Outlines of Human Pathology. By HERBERT MAYO, F.R.S. .. .. .. .. .. } 1
Illustrations of the Elementary Forms of Disease. By ROBERT CARSWELL, M.D. .. }

1. Hypertrophy of Bone .. .. .. .. .. .. .. .. .. .. .. .. 1
2. Atrophy of Bone.. .. .. .. .. .. .. .. .. .. .. .. .. 5
3. Simple Inflammation of Bone .. .. .. .. .. .. .. .. .. .. 7
4. Abscess of Bone .. .. .. .. .. .. .. .. .. .. .. .. 7
5. Necrosis .. .. .. .. .. .. .. .. .. .. .. .. .. 8
6. Caries . .. .. .. .. .. .. .. .. .. .. .. .. .. 9
7. Malignant Growths .. .. .. .. .. .. .. .. .. .. .. .. 12
8. Medullary Sarcoma .. .. .. .. .. .. .. .. .. .. .. .. 13
9. Hydatids in Bone .. .. .. .. .. .. .. .. .. .. .. .. 15
10. Diseases of the Joints .. .. .. .. .. .. .. .. .. .. .. 16
11. Synarthroses .. .. .. .. .. .. .. .. .. .. .. .. .. 16
12. Diarthroses .. .. .. .. .. .. .. .. .. .. .. .. .. 17
13. Diseases of the Bursæ Mucosæ .. .. .. .. .. .. .. .. .. 27
14. Dr. Carswell on Pus .. .. .. .. .. .. .. .. .. .. .. .. 28

II.

Statistical Inquiry into the present State of the Medical Charities in Ireland, &c. By Mr. PHELAN.. .. .. .. .. .. .. .. .. .. .. .. .. .. 34

III.

A further Inquiry concerning Constitutional Irritation, and the Pathology of the Nervous System. By BENJAMIN TRAVERS, Esq. F.R.S. .. .. .. . .. 37

IV.

Practical Observations on the Nature and Treatment of Nervous Diseases. By Mr. MART 49

1. Paralytic Affections .. .. .. .. .. .. .. .. .. .. .. .. 49
2. Amaurosis .. .. .. .. .. .. .. .. .. .. .. .. .. 53
3. Nervous Indigestion .. .. .. .. .. .. .. .. .. .. .. .. 54
4. Tic Douloureux .. .. .. .. .. .. .. .. .. .. .. .. 55
5. Neuralgia .. .. .. .. .. .. .. .. .. .. .. .. .. 56

V.

Experimental Researches on the Ganglionic System. By M. BRACHET .. .. .. 57

1. General Functions .. .. .. .. .. .. .. .. .. .. .. .. 60
2. Special Functions of the Ganglionic Nerves .. .. .. .. .. .. .. 67
3. Influence of the Brain on the Heart .. .. .. .. .. .. .. .. 68
4. Influence of the Spinal Marrow on the Heart .. .. .. .. .. .. 70
5. Influence of the Pneumogastric on the Heart .. .. .. .. .. .. 71
6. Influence of the Great Sympathetic .. .. .. .. .. .. .. .. 73

VI.

Mr. LEE on the Medical Institutions of France, Germany, and Italy .. .. .. .. 75

VII.

Mr. KIRBY on the Power, Wisdom, and Goodness of God, in the Creation of Animals, 79

VIII.

Medico-Chirurgical Transactions. Vol. XIX. 1835.

1. On Pulmonary Phlebitis. By Dr. ROBERT LEE .. .. .. . .. .. 94
2. Singular Laceration of the Peritoneal Coat of the Uterus. By W. W. PARTRIDGE, Esq. .. .. .. .. .. .. .. .. .. .. .. .. 95

3. Observations on Fibro-calcareous Tumors in the Uterus. By Dr. R. Lee 96
4. Cases of Warty Tumors in Cicatrices. By Cæsar Hawkins, Esq. .. .. 98
5. Cases of Fracture of the Neck of the Femur. By J. Howship, Esq. .. .. 102
6. Observations on Fractures of the Bones of the Pelvis. By Henry Earle, Esq. 107
7. Cases and Observations illustrating the Diagnosis of Peritoneal Adhesions. By Richard Bright, M.D. .. .. .. .. .. .. .. .. .. .. .. .. 110
8. On Affections of the Brain. By Dr. Sims .. .. .. .. .. .. .. 117
   1. Serous Effusion .. .. .. .. .. .. .. .. .. .. .. .. .. 119
   2. Hypertrophy and Atrophy of the Brain .. .. .. .. .. .. .. 125
   3. Ramollissement of the Brain .. .. .. .. .. .. .. .. .. 135
9. Cases of Mental Derangement, successfully treated by Acetate of Morphia. By Dr. Seymour .. .. .. .. .. .. .. .. .. .. .. .. 141

IX.

Narrative of a Voyage round the World, &c. By Dr. Wilson .. .. .. .. .. 143
Disasters by Shipwreck .. .. .. .. .. .. .. .. .. .. .. .. 144
Remarkable Plasticity of the Human Frame under Privation and Sufferings, illustrated by Ancient and Modern Instances .. .. .. .. .. .. .. 145

X.

Practical Observations on Diseases of the Heart, Lungs, Stomach, Liver, &c. occasioned by Spinal Irritation. By John Marshall, M.D. .. .. .. .. .. .. .. 146

XI.

On Dropsies connected with suppressed Perspiration and coagulable Urine. By Dr. J. Osborne .. .. .. .. .. .. .. .. .. .. .. .. .. .. .. 158

### PERISCOPE.

PART I.

### Spirit of the English Periodicals, and Notices of English Medical Literature.

1. Fatal Abdominal Disease of an insidious nature. By Dr. J. Johnson .. .. .. 161
2. Paracentesis Thoracis in Hydrothorax—at the Lowestoft Infirmary.. .. .. . 162
3. Remarkable Case of the Proteian Malady—Irritation simulating Inflammation .. 163
4. On Puncture of the Bladder above the Pubes. By Dr. Geddes .. .. .. .. 166
5. On Diseases of the Stomach—their Sympathies and Complications. By Mr. L. Parker 167
6. Dr. Aldis on Pulmonary Apoplexy .. .. .. .. .. .. .. .. .. .. 171
7. On Anæmia. By Dr. Geddes .. .. .. .. .. .. .. .. .. .. .. 172
8. Dr. M'Leod on Intestinal Convulsions .. .. .. .. .. .. .. .. 173
9. Provincial Medical Education .. .. .. .. .. .. .. .. .. .. 173
10. On Scarlatina. By Dr. Sandwith .. .. .. .. .. .. .. .. .. 175
11. Dr. Thorburn on the Elements of Clinical Medicine .. .. .. .. .. .. 177
12. Dr. Davis on the Induction of Premature Labour .. .. .. .. .. .. 178
13. On Pterygium. By Mr. Middlemore.. .. .. .. .. .. .. .. .. 179
14. Case of great Enlargement of the Articular Epiphyses from Rickets. By T. Brayne, Esq. .. .. .. .. .. .. .. .. .. .. .. .. .. .. 182
15. Remarkable Disease of the Stomach and Colon—Total Occlusion of the latter for 25 Days. By James Johnson, M.D. .. .. .. .. .. .. .. .. 184
16. Desperate Wound in the Neck—Ligature of the Carotid—Recovery. By Mr. Tierman .. .. .. .. .. .. .. .. .. .. .. .. .. .. .. 187
17. Scandinavian Contributions to Practical Medicine.
   1. On the Employment of Venesection and Antimony in Intermittents. By Dr. Neverman .. .. .. .. .. .. .. .. .. .. .. .. .. 189
   2. On the same Remedies by Dr. Westergard .. .. .. .. .. .. .. 190
   3. Effects of Blisters in stopping Hiccup .. .. .. .. .. .. .. 192
   4. Empyema of the left Pleural Cavity, attended by peculiar circumstances .. 192
18. Remarks on Malformations of the internal Ear—the Result of Post-mortem Examinations. By Mr. Cock.. .. .. .. .. .. .. .. .. .. .. 194
19. Examination of the Organs of Hearing, from the Body of a Boy, the subject of Congenital Deafness. By Mr. Thurnham .. .. .. .. .. .. .. 196
20. Cases in Surgery. By Professor Smith, of Maryland.
   1. Calculus in the Bladder—Sloughing of the Glans Penis .. .. .. .. .. 197

2. Large Stones broken in the Bladder .. 198
3. Lithotomy—Secondary Hæmorrhage .. 199
4. Aneurismal Varyx—Ligature of the Brachial Artery .. 200
21. Remarkable Disease of the Brain, attended with unusual and distressing Phenomena. By James Johnson, M.D. .. 202
22. Case of Salivation from Iodine. By Dr. Mackall, of Maryland .. 203
23. Identity of Gonorrhœa and Syphilis. By Professor Hufeland, with Strictures .. 204
24. On Venereal Affections of the Testis. By Dr. Cusack, with Observations .. 205
25. Phrenology. Case of supposed impairment of the Faculty of Language .. 207
26. Phrenological Quacks .. 208

### Spirit of the Foreign Periodicals, &c.

1. On Monstrosities in General .. 209
2. Lateral Transposition of the Viscera—Great Cardiac Malformation without Cyanosis 215
3. Obliteration of the Aorta, Common Iliacs, &c. .. 217
4. Obliteration of the Vena Cava .. 219
5. On the Auscultatory Signs of Pregnancy. By Dr. Hohl .. 220
1. Changes in the Two Sounds during Labour .. 224
2. Tabular View of the Pulse of the Fœtus. .. 228
6. Cases of a Milky State of the Blood .. 229
7. On the Chemical Condition of the Saliva, as an Indication of the different Morbid Affections of the Stomach. By M. Donné .. 231
8. Small Pox at Naples—Dr. Gregory and others on the late Epidemic in London—Reciprocal Effects of Variola and Vaccinia .. 234
9. Disease of the Uterus, benefited by Hydriodate of Potash—Dr. Clendinning's Observations on this Medicine .. 241
10. On the Diseases of the Lymphatic System .. 244
11. State of the Lymphatics in Puerperal Fever—from the French of Velpeau and Others .. 245
12. On the Use of Local Applications to the Pustules of Variola. From the French of Velpeau, Bretonneau, Guersent, Serres, Damiron, &c. .. 249
13. On the Symptoms and Treatment of Gall-stones, with Cases and Observations.
1. Grief of Mind—repeated Attacks of Jaundice .. 251
2. Mental Distress—Epigastric Pain .. 251
3. Repeated Attacks of Epigastric Pain, &c. .. 252
4. Vomiting of Biliary Calculi .. 252
5. On the Symptoms of Biliary Calculi .. 253
14. Case of Mollities Ossium, and Fatty Degeneration of the Muscles .. 254
15. Hydatid Cyst opening into the Intestines and Urinary Bladder. Remarks on Hydatids in General .. 255

### Clinical Review and Hospital Reports.

### County of Meath Hospital.

1. Preliminary Remarks on Inductive Reasoning and Hypothetical Speculations .. 257
Cases of Aneurism .. 257
2. Aneurism of the Posterior Tibial Artery .. 258
3. Diffused False Aneurism of Popliteal Artery .. 259
4. Circumscribed False Aneurism of Brachial Artery .. 259
5. Aneurismal Varix at the Bend of the Arm .. 261

### St. Thomas's Hospital.

Syphilitic Eruptions treated with Hydriodate of Potass .. 262
6. Roseola Syphilitica .. 262
7. Purpura Syphilitica .. 264
8. Rupia Syphilitica .. 264
9. Syphilitic Lichen .. 265

### St. George's Hospital.

### Pathology of Continued Fever.

10. Preliminary Remarks on this Subject .. 266
11. Continued Fever—Abdominal Tenderness .. 267
12. Continued Fever—Hæmatirrhœa .. 267
13. Continued Fever—Furuncular Eruption .. 268
14. Chronic Peritonitis—Perforation .. 269

PENNSYLVANIA HOSPITAL.

Cases of Dislocation and Fracture .. .. .. .. . .. . . .. .. .. 271

15. Dislocation of the Head of the Os Femoris .. .. .. .. .. .. .. .. .. 271

16. Case of Mr. Keate's in Illustration .. .. .. .. .. .. .. .. .. .. .. 272

17. Odd Union of Fractured Patella .. .. .. .. .. .. .. .. .. .. .. .. 273

18. Fracture of External Condyle of Femur .. .. .. .. .. .. .. .. .. .. 273

20. Fracture of Humerus from Muscular Exertion .. .. .. .. .. .. .. .. 274

Injuries of the Head .. .. .. .. .. .. .. .. .. .. .. .. .. .. .. 274

21. Wounds of the Scalp .. .. .. .. .. .. .. .. .. .. .. .. .. .. 274

22. Fracture with Depression .. .. .. .. .. .. .. .. .. .. .. .. .. 274

CLINICAL LECTURES.

23. Mr. Travers on Clinical Instruction .. .. .. .. .. .. .. .. .. .. .. 276
24. Clinical Lectures of Sir B. Brodie .. .. .. .. .. .. .. .. .. .. .. 278
25. On Epulis .. .. .. .. .. .. .. .. .. .. .. .. .. .. .. .. .. 278
26. On Affections of the Rectum—Contraction of Sphincter Ani.. .. .. .. .. .. 281
27. Ulcer of the Rectum .. .. .. .. .. .. .. .. .. .. .. .. .. .. .. 281
28. Stricture of the Rectum .. .. .. .. .. .. .. .. .. .. .. .. .. .. 282
29. Mr. Green on Employment of Seton for Hydrocele .. .. .. .. .. .. .. .. 283
30. Mr. Travers on Absorption of the Cranium .. .. .. .. .. .. .. .. .. 285
31. Mr. Guthrie on the Removal of large Calculi .. .. .. .. .. .. .. .. .. 287

**Miscellanies.**

1. The Cyclopædia of Anatomy and Physiology .. .. .. .. .. .. .. .. .. 289
2. Death after Morrison's Pills—Coroner's Inquest—Dissection.. .. .. .. .. .. 290
3. Case of Aggravated Purpura, or rather Carditis, attended with unusual Circumstances. By Dr. Murray of Trinidad .. .. .. .. .. .. .. .. .. .. 291
4. Description of a most Virulent and Fatal Epidemic in Yorkshire. By Mr. R. Howard 292
5. Case of Fractured Ribs—Rupture of the Liver—Formation of Biliary Calculi in the Lacerated Parts, &c. By Dr. Fazely, of Trinidad.. .. .. .. .. .. .. 296

**Obituary.**

1. Death of Sir David Barry, with the Appearances on Dissection .. .. .. .. .. 298
2. Death of Dr. Warren, with Sketch of his Character, &c. .. .. .. .. .. .. 300
3. Death of Professor Turner .. .. .. .. .. .. .. .. .. .. .. .. .. 301
4. Bibliographical Record.. .. .. .. .. .. .. .. .. .. .. .. .. .. 302

